# Screening of Oral Potentially Malignant Disorders Using Exfoliative Cytology: A Diagnostic Modality

**DOI:** 10.1155/2016/8134832

**Published:** 2016-09-18

**Authors:** Arpita Kabiraj, Tanya Khaitan, Debarati Bhowmick, Uday Ginjupally, Aritri Bir, Kushal Chatterjee

**Affiliations:** ^1^Department of Oral Pathology & Microbiology, Haldia Institute of Dental Sciences and Research, Haldia, West Bengal 721645, India; ^2^Department of Oral Medicine & Radiology, Haldia Institute of Dental Sciences and Research, Haldia, West Bengal 721645, India; ^3^Department of Periodontics, Haldia Institute of Dental Sciences and Research, Haldia, West Bengal 721645, India; ^4^Department of Oral Medicine & Radiology, Kamineni Institute of Dental Sciences, Narketpally, Andhra Pradesh 508254, India; ^5^Department of Biochemistry, IQ City Medical College, Durgapur, West Bengal 713206, India; ^6^Department of Dentistry, Jawaharlal Nehru Medical College & Hospital, Kalyani, West Bengal 74123, India

## Abstract

*Objective*. Oral exfoliative cytology (OEC) has been implemented in the diagnosis of pathologic lesions for ages. The present study was undertaken to evaluate the cytomorphological features of some of the commonest potentially malignant disorders (leukoplakia, lichen planus, and oral submucous fibrosis) through a simple procedure and illustrate its importance in mass screening.* Materials and Method*. A total of 160 subjects with 25–50 years of age were included in the study. Among them, 40 were clinically diagnosed with oral leukoplakia, 40 were diagnosed with oral lichen planus, 40 were diagnosed with oral submucous fibrosis, and 40 were in the control group. The prepared smears were subjected to Papanicolaou stain and analyzed microscopically for the evaluation of the cytomorphological features.* Results and Discussion*. When analyzed microscopically, 36 (90%) out of the 40 oral leukoplakic lesions showed Class II cytological features whereas 4 (10%) revealed Class I features. Among 40 patients with oral lichen planus, 26 (65%) showed Class II features while the remaining 14 (35%) revealed Class I features. In 40 subjects with oral submucous fibrosis, 32 (80%) showed Class II features while the other 8 (20%) showed Class I features. All the 40 control subjects showed Class I features. Thus, OEC can be widely advocated as an addition to clinical conclusion and an adjunct to biopsy.

## 1. Introduction

Oral mucosa exhibits a rapid turnover of cells and these exfoliated cells play an imperative role in diagnosis of potentially malignant disorders. Oral cavity reflects the various events occurring in the body which is revealed by the cytomorphological and nucleomorphological variations in these exfoliated cells [1].

Oral exfoliative cytology (OEC) is the microscopic examination of exfoliated cells from an epithelial surface. It is a simple, noninvasive, and sensitive staining technique used as an adjuvant for biopsy or in cases where biopsy is not feasible as well as mass screening [2].

Literature suggests that oral cytology may be helpful for detecting potentially malignant disorders or early carcinoma in asymptomatic patients with lesions that appear benign. Early detection of such lesions increases the endurance and decreases the morbidity of such patients. The features of cytological atypia usually observed in such disorders include cellular and nuclear pleomorphism, nuclear budding, hyperchromatism and micronuclei, inflammatory cells, indented cellular outline, and intracytoplasmic vacuoles [3]. Considering this background, the present study was undertaken to evaluate the cytomorphological features of some of the commonest potentially malignant disorders through a simple exfoliative cytology procedure and also exemplify its importance in mass screening.

## 2. Materials and Method

The study was initiated after the protocol had been approved by the Institutional Ethical Committee. A total of 160 subjects belonging to the age group 25–50 yrs were included in the study. Among them, 40 were clinically diagnosed with oral leukoplakia, 40 with oral lichen planus, and 40 with oral submucous fibrosis. 40 subjects with no history of habits and no abnormal clinical features on examination were included in the control group. Subjects with history of any systemic illness were excluded from the study.

The importance and need for the study were explained and an informed consent was obtained from all the individuals participating in the study. Buccal smears were obtained from the lesional area using a wooden spatula through a simple conventional technique from all the subjects. The smears were then prepared on the slides which were subjected to Papanicolaou stain (Rapid PAP kit) and analyzed microscopically for the evaluation of the cytomorphological features. They were classified according to Papanicolaou's classification (1960) as follows: Class I (normal): only normal cells observed; Class II (atypical): presence of minor atypia but no evidence of malignancy; Class III (intermediate): an in-between cytology (the cells display wide atypia that may be suggestive of malignancy but are not clear cut cancer and represent precancerous lesions or in situ carcinoma); Class IV (suggestive of cancer): a few epithelial cells with malignant characteristics or cells with borderline characteristics; Class V: positive cancer cells that are obviously malignant [4].

## 3. Results

**Figure 1:**
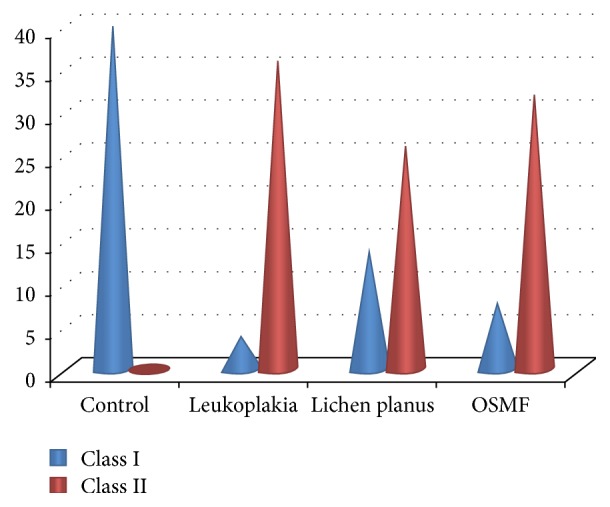
Graphical presentation of Class I and Class II features in oral leukoplakia, lichen planus, oral submucous fibrosis, and control.

**Figure 2:**
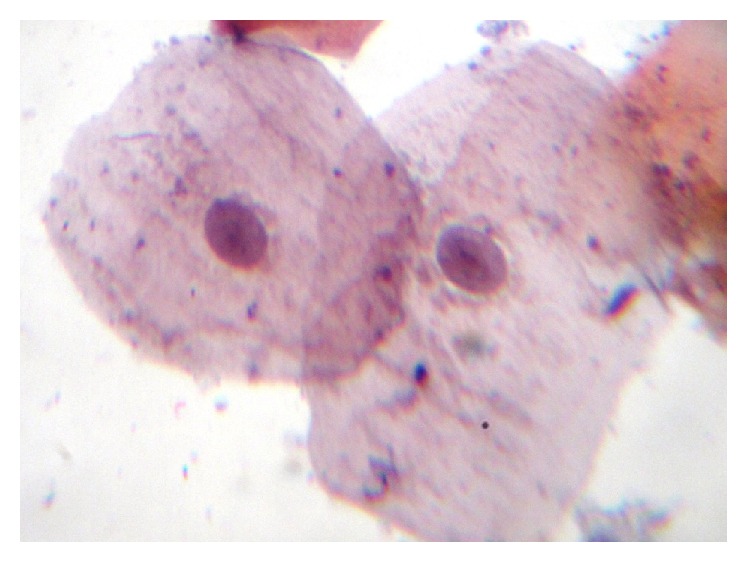
Photomicrograph showing epithelial cells with nuclear pleomorphism, prominent nucleoli, hyperchromatism, and micronuclei (40x).

**Figure 3:**
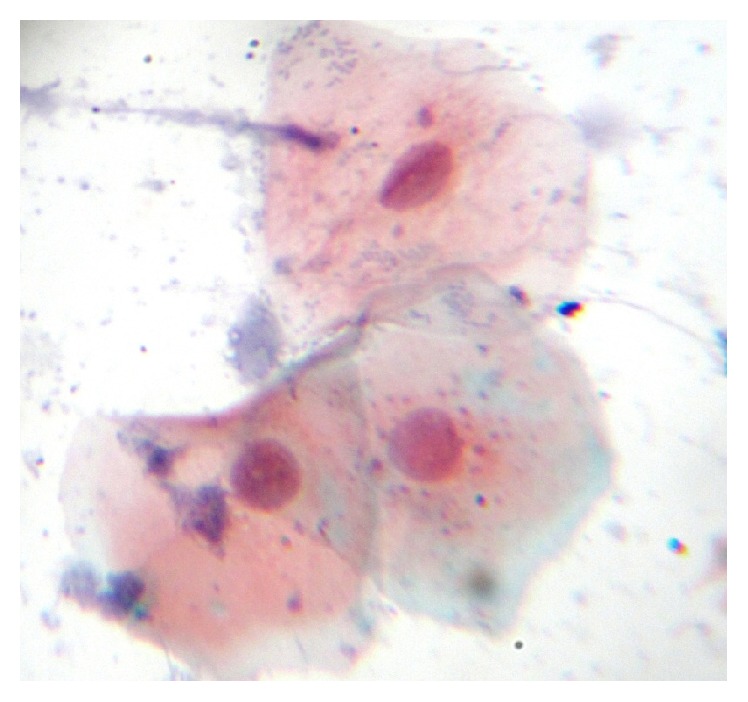
Photomicrograph showing epithelial cells with enlarged hyperchromatic nuclei and intracytoplasmic vacuoles (40x).

**Figure 4:**
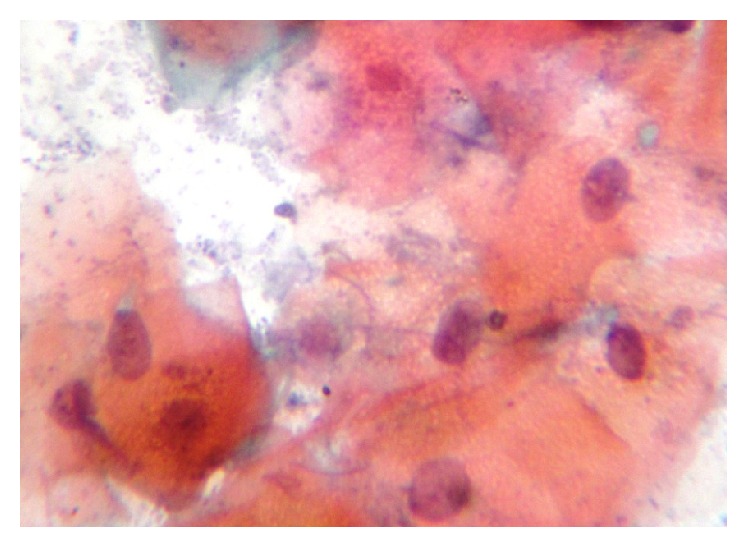
Photomicrograph showing epithelial cells clumping of chromatin within the nucleus (40x).

**Figure 5:**
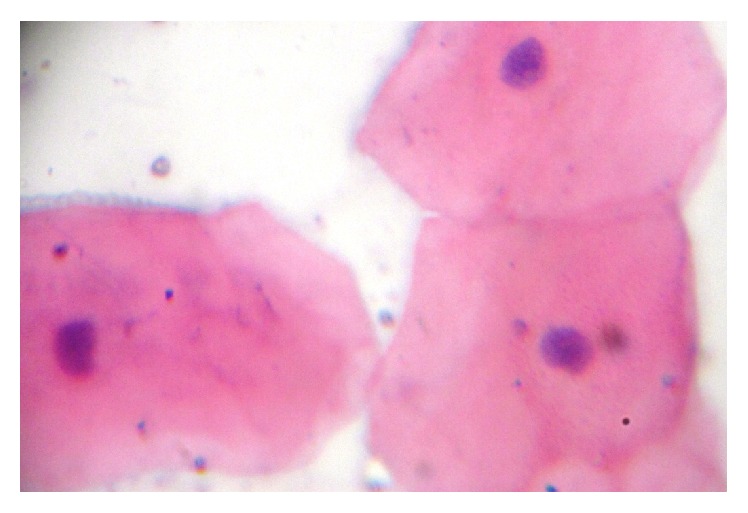
Photomicrograph showing epithelial cells with normal cellular and nuclear morphology (40x).

Among 160 individuals, 40 were clinically diagnosed with oral leukoplakia, 40 were with oral lichen planus, 40 were with oral submucous fibrosis, and 40 were control. When analyzed microscopically, 36 (90%) out of the 40 oral leukoplakic lesions showed Class II cytological features whereas 4 (10%) revealed Class I features. Among 40 patients with oral lichen planus, 26 (65%) of them showed Class II features while the remaining 14 (35%) revealed Class I features. In 40 subjects with oral submucous fibrosis, 32 (80%) showed Class II features while the other 8 (20%) showed Class I features. All the 40 control subjects revealed Class I features [Figure 1]. Class II cytological features observed were cellular and nuclear pleomorphism, irregular cellular outline, perinuclear halo, free nuclei, and both intranuclear and intracytoplasmic vacuolization along with numerous bacterial colonies. Additionally, cells showed inflammatory changes like indented cellular outline and intracytoplasmic vacuoles suggestive of cells undergoing autolysis [Figures 2, 3, and 4]. Class I cytological features showed exfoliated cells of normal size and shape [Figure 5].

## 4. Discussion

Oral cavity is susceptible to countless changes with advancing, environmental, and lifestyle related habits and factors. Oral mucosal lesions especially related to chewing and smoking of tobacco have led to the increased incidence and prevalence of potentially malignant and malignant disorders worldwide. OEC is the microscopic examination of exfoliated cells from epithelial surface. Papanicolaou and Traut's staining technique for cytology smears was first used in oral leukoplakia by Montgomery and von Hamm [2]. The incidence of oral potentially malignant disorders is high in India and its subcontinents. Literature reveals that the prevalence of oral leukoplakia varies from 0.2% to 5.2% with malignant transformation of 0.13% and 10% in India [5]. The prevalence of oral lichen planus has been reported to be 0.1 to 1.5% while being 0.03% to 3.2% for OSMF, which is gradually increasing owing to the excessive usage of areca nut and tobacco products among various groups of population [6]. Quantitative cytomorphometric assessment of the exfoliated buccal mucosal cells has shown measurable changes in cells obtained from potentially malignant and malignant disorders. Moreover, OEC offers a simple nonaggressive technique that can be repeated frequently with little discomfort to the patient and better compliance [7].

Our study was therefore carried out to assess the cytomorphometric features of cells obtained from buccal scrapings in some of the commonest oral potentially malignant disorders and to employ these features to detect dysplasia and malignancy in their early stages [8]. In the present study, it was observed that Class I cytologic features were evident in 35% oral lichen planus, 10% leukoplakia, 20% oral submucous fibrosis cases, and all controls. However, Class II features were seen in 65% oral lichen planus, 90% leukoplakia, and 80% oral submucous fibrosis cases. The features of cytological atypia that were recorded in the present study included cellular pleomorphism, nuclear pleomorphism, nuclear budding, hyperchromatism and micronuclei, bacterial colonies, inflammatory cells and indented cellular outline, and intracytoplasmic vacuoles indicative of cytolysis.

Although the application of OEC for identifying potentially malignant and malignant lesions has been debatable, cytologic smears were useful for diagnosing leukoplakia and oral submucous fibrosis lesions in their early stages. Previous studies performed on OEC have concluded that the technique is useful in lesions of leukoplakia and oral submucous fibrosis. It has been known to be a useful adjunct that reflects early epithelial dysplasia in the development of the experimental tumor and for diagnosing very early malignant change [3]. Lichen planus being more prevalent in our study population was also included along with leukoplakia and OSMF as the malignant transformation of lichen planus is seen to be at a higher rate. Our study subjects were also being compared with the control group which was not in accordance with previous studies.

In a study from Sudan, cytological analysis of buccal scrapings has been proposed as a useful early diagnostic method for epithelial atypia and malignant oral lesions where nearly 5% of clinically benign appearing mucosal lesions were sampled by this technique and later confirmed by typical scalpel biopsy to represent dysplastic epithelial changes or invasive cancer [9]. Singh (2010) elucidated the role of exfoliative cytology in determining the cellular atypical features of oral leukoplakia and oral submucous fibrosis [4]. Kumar et al. (2011) observed 69% sensitivity in leukoplakia cases using OEC [2]. Earlier cytomorphometric studies suggested that a decrease in the mean cytoplasmic diameter of exfoliated buccal mucosal cells could serve as an early indicator of dysplastic change, especially in lesions which appear histologically. Later, it was suggested that such diagnosis should be used to help identify patients at increased risk of developing cancer. Micronucleus refers to the small nucleus that forms whenever a chromosome or a fragment of a chromosome is not incorporated into one of the daughter nuclei during cell division. These may serve as marker for increased cancer risk, since they have been reported to arise in response to DNA damaging agents. Micronuclei are found at increased frequencies from normal mucosa to potentially malignant disorders to carcinoma, especially in the head and neck region suggesting ever increasing chromosomal instability [10].

Early oral potentially malignant disorders and cancers often are understated and asymptomatic. On occasion, certain histopathologic changes may arise in areas where there is no clinical evidence of any lesion. Therefore, it is imperative for a clinician to take into account the suspicious elements, especially if risk factors such as tobacco or alcohol use are involved. Atypical and dysplastic cells show a significant increase in nuclear area and diameter due to the increased nuclear content required for replication; the ability of such cells to form cytoplasm is decreased. Therefore, in malignant cells the nuclear dimensions increase and the overall cellular dimensions decrease. The diagnostic efficacy of OEC depends on the impression that changes in the superficial cells do imitate changes occurring in the underlying tissue.

OEC is widely advocated as an addition to clinical conclusion and an adjunct to biopsy. The smear technique is not intended to replace tissue biopsy but can be valuable and useful for detecting early malignant changes or recurrence, where biopsy is contraindicated or in cases of postradiotherapy follow-up. It certainly promises to improve the survival rate of patients suffering from such conditions. With OEC being an easy, noninvasive, cost-effective technique, cytomorphometric analysis of exfoliated cells could be done for mass screening and regular follow-up of potentially malignant disorders. However, further studies should be conducted on a larger population to establish the role of OEC in potentially malignant disorders.
